# Obesity and Risk of Peptic Ulcer Disease: A Large-Scale Health Check-Up Cohort Study

**DOI:** 10.3390/nu11061288

**Published:** 2019-06-06

**Authors:** Jeung Hui Pyo, Hyuk Lee, Jee Eun Kim, Yoon Ho Choi, Tae Jun Kim, Yang Won Min, Byung Hoon Min, Jun Haeng Lee, Poong Lyul Rhee, Heejin Yoo, Kyunga Kim, Jae J. Kim

**Affiliations:** 1 Center for Health Promotion, Samsung Medical Center, Sungkyunkwan University School of Medicine, Seoul 06351, Korea; jeunghui.pyo@samsung.com (J.H.P.); eustina.kim@samsung.com (J.E.K.); yh38.choi@samsung.com (Y.H.C.); 2 Department of Medicine, Samsung Medical Center, Sungkyunkwan University School of Medicine, Seoul 06351, Korea; tj23.kim@samsung.com (T.J.K.); yangwon.min@samsung.com (Y.W.M.); jason.min@samsung.com (B.H.M.); jh2145.lee@samsung.com (J.H.L.); pl.rhee@samsung.com (P.L.R.); jaej.kim@samsung.com (J.J.K.); 3 Statistics and Data Center, Research Institute for Future Medicine, Samsung Medical Center, Seoul 06351, Korea; heejin17.yoo@sbri.co.kr (H.Y.); kyunga.j.kim@samsung.com (K.K.)

**Keywords:** Obesity, Metabolically healthy obesity, Peptic ulcer disease, Gastric ulcer, Duodenal ulcer

## Abstract

The association between obesity and peptic ulcer disease (PUD) is inconclusive. To evaluate the association of obesity and metabolically healthy obesity (MHO) with PUD risk, we performed a retrospective cohort study of 32,472 subjects without PUD at baseline who underwent repeated health examinations. Participants were stratified by body mass index (BMI) and metabolically healthy state. Hazard ratios (HRs) and 95% confidence intervals (CIs) were calculated using Cox proportional hazard modelling. During the follow-up period, 1940 PUD cases occurred. PUD, particularly gastric ulcer (GU), had significantly higher cumulative incidence in obese subjects compared to non-obese subjects (*p* value < 0.001). The HR for developing GU was 1.32 (95% CI, 1.16–1.49; *p* value <0.001); after adjusting for confounding factors (lifestyle, metabolic, and *Helicobacter pylori* status), the association was no more significant (*p* value = 0.789). For duodenal ulcer (DU), cumulative incidence between obese and non-obese groups was not significantly different (*p* value = 0.464). The risk of developing DU in the obese group was not significantly different from the non-obese group (HR 0.95; 95% CI, 0.83–1.09; *p* value = 0.469) and consistently showed no association after adjusting for metabolic parameters (*p* value = 0.199). Furthermore, MHO subjects had no increase in GU or DU risks. In this large cohort study, PUD risk was not associated with obesity or MHO.

## 1. Introduction

Despite the decreasing incidence of peptic ulcer disease (PUD) and its complications due to the discovery of potent acid suppressants and eradication of *Helicobacter pylori* infection, PUD remains one of the most commonly encountered gastrointestinal diseases with a significant impact on the healthcare system [1,2].

The prevalence of idiopathic ulcer, ulcers not caused by *Helicobacter pylori* infection or nonsteroidal anti-inflammatory drugs (NSAIDs) or aspirin, is increasing [3] and past studies proposed possible risk factors [4,5]. Obesity has been reported as a risk factor of PUD in many studies [4,5,6,7,8], yet conflicting results have been reported in several studies [9]. It is unclear whether PUD is independently associated with obesity. Moreover, recently, interest has been focused on the metabolically healthy obese (MHO), subjects who have a unique subset of characteristics, which reduce metabolic and cardiovascular risk factors despite excess adiposity [10,11]. However, no study has evaluated the role of MHO as a determinant of PUD.

Therefore, we sought to investigate if obese and MHO subjects have greater PUD risk among a large cohort of subjects who participated in a health screening examination program with detailed information on lifestyle factors while controlling for potential confounder.

## 2. Materials and Methods

### 2.1. Study Population and Design

We conducted a retrospective cohort study based on the population of men and women aged 20 years or older who underwent a comprehensive annual or biannual health examination at the Centre for Health Promotion, Samsung Medical Centre, Seoul, Korea, between January 2005 and June 2017. Since our study aim was to evaluate the association between obesity and the risk of developing PUD, the study population was restricted to subjects who enrolled in the health check-up program and underwent at least two screening exams including esophagogastroduodenoscopy (EGD) taken at least 1 year apart (*n* = 230,794). We excluded participants who had PUD on EGD at baseline (*n* = 17,542), self-reported history of malignancy (*n* = 5437), regular use of NSAIDs, antiplatelet, or anticoagulant (*n* = 18,899), malignant ulcer (*n* = 785), and missing data on important covariates (anthropometry, metabolic parameters, or EGD) (*n* = 69,476).

This comprehensive health-screening program included a questionnaire on lifestyle factors, medication use, and chronic disease; physical examinations; and a series of laboratory, radiologic, and endoscopy examinations. All examinations were performed and diagnosed by medical specialists. Subjects paid voluntarily for their health check-ups while others were partly supported by affiliated companies. The study protocol was approved by the institutional review board of Samsung Medical Centre, and because of the retrospective nature of the study, the requirement for informed consent was waived.

### 2.2. Assessment of Obesity and MHO

Obesity was defined according to Asia-Pacific body mass index (BMI) criteria (non-obese <25 kg/m^2^ and obese ≥25 kg/m^2^), which are defined by the World Health Organization [12]. MHO was defined according to Wildman criteria as having less than two of the following risk factors [13]; (1) systolic blood pressure ≥130 mmHg and/or a diastolic blood pressure ≥85 mmHg, or on antihypertensive treatment; (2) triglyceride ≥150 mg/dL (1.7 mmol/L); (3) fasting plasma glucose ≥100 mg/dL (5.6 mmol/L) or on anti-diabetic treatment; (4) high-density lipoprotein (HDL) cholesterol <40 mg/dL (1.0 mmol/L) in men, <50 mg/dL (1.3 mmol/L) in women; (5) Homeostatic model assessment-insulin resistance (HOMA-IR) >90th percentile (>2.84); (6) High-sensitivity C-reactive protein (hsCRP) level >90th percentile (>0.22 mg/dL).

Four subgroups regarding obesity and metabolic health status were created: (1) metabolically healthy, non-obese (MHNO): BMI <25 kg/m^2^ and <2 metabolic risk factors; (2) metabolically unhealthy, non-obese (MUNO): BMI <25 kg/m^2^ and ≥2 metabolic risk factors; (3) MHO: BMI ≥25 kg/m^2^ and <2 metabolic risk factors; or (4) metabolically unhealthy, obese (MUO): BMI ≥25 kg/m^2^ and ≥2 metabolic risk factors.

### 2.3. Assessment of EGD for PUD

Endoscopy was performed by 27 board-certified gastroenterologists using Olympus GIF XQ 260 and GIF Q240 or 260 (Olympus Medical Systems Corp, Tokyo, Japan) after overnight fasting. Peptic ulcer was defined as a circumscribed mucosal break 5 mm or more in diameter, with a well-defined ulcer crater [14]. The peptic ulcer was staged, by use of the endoscopic staging system of Sakita, into three stages (active, healing, and scarring). The *Helicobacter pylori* status was detected histologically by means of haematoxylin and eosin staining in all subjects who underwent histological studies. Biopsy specimens were taken from lesions suspected to be major gastric findings or for the purpose to detect the presence of *Helicobacter pylori*.

### 2.4. Assessment of Covariates

Subjects filled out a standard questionnaire regarding their personal medical history (including hypertension and diabetes), medications (including regular use of aspirin, NSAIDs, or other anti-inflammatory analgesics) and health-related behaviors; smoking status was categorized as never, former, and current. Heavy alcohol intake was defined as ≥20 g/day (heavy drinking). Regular exercise was defined as exercising three or more times per week with moderate intensity physical activity.

Participants′ physical measurements were obtained, and their serum biochemical parameters were analyzed and determined by hospital trained staff. BMI [weight (kg)/height (m)^2^] was calculated and categorized according to the WHO Western Pacific Region [12]. Waist circumference was measured at the midpoint between the inferior margin of the last rib and the superior iliac crest in a horizontal plane. The blood chemistry tests were performed after the individuals had fasted for 12 h. Total cholesterol, low-density lipoprotein (LDL) and HDL cholesterol, triglyceride, and fasting blood glucose levels were measured using enzymatic or colorimetric methods (Hitachi Ltd, Tokyo, Japan). Serum insulin level was measured by immunoradiometric assay (Biosource, Belgium). hsCRP level quantitation was done using the immunonephelometry method (Behring, Nephelometer II, Germany). HOMA-IR was calculated as fasting insulin (μU/L) × fasting glucose (mg/dL)/405 [15].

### 2.5. Statistical Analysis

Statistical analysis was executed using SAS version 9.4 (SAS Institute Inc., Cary, NC, USA) and R 3.4.3 (Vienna, Austria). We determined baseline frequencies for demographic characteristics. Continuous variables were expressed as mean ± standard deviation (SD), while categorical variables were expressed as proportions (%). We compared the subjects in the two groups using the Wilcoxon rank sum test and the Chi-squared test. Cumulative incidence was calculated using Kaplan–Meier curves. The association of risk factors with outcomes was identified by Cox proportional-hazards regression models. Multicollinearity was checked using variance inflation factor (VIF). There are no variables with VIF >4. We calculated hazard ratios (HRs) with 95% confidence intervals (CIs) for developing PUD. A *p* value <0.05 was considered statistically significant. We used four models with increasing degrees of adjustments to account for potential confounding factors at baseline. Model 1 was adjusted for age and sex. Model 2 was further adjusted for variables in Model 1, plus health-related behaviors and comorbidity including alcohol intake, smoking, and physical activity. Model 3 was further adjusted for variables in Model 2, plus metabolic variables including blood pressure, use of antihypertensive medication, fasting blood plasma, use of hypoglycemic medications, triglyceride, HDL-cholesterol, and LDL-cholesterol. Model 4 was further adjusted for variables in Model 3, plus *Helicobacter pylori* status.

## 3. Results

### Baseline Characteristics

Overall 32,472 subjects were included in the study. Clinical and biochemical characteristics of the study subjects according to obesity status are shown in Table 1. The mean (SD) age and BMI of the study participants at baseline was 50.2 (8.0) years and 23.6 (3.0) kg/m^2^, respectively. The prevalence of obesity was 31.3%. Compared to non-obese subjects, obese subjects were more likely to be male, slightly older, current smokers, and heavy drinkers; and more likely to exercise regularly, have higher levels of total cholesterol, LDL-cholesterol, triglyceride, fasting blood glucose, insulin, HOMA-IR, hsCRP; and lower level of HDL-cholesterol.

## 4. Cumulative Incidence of PUD among Subjects

**Figure 1:**
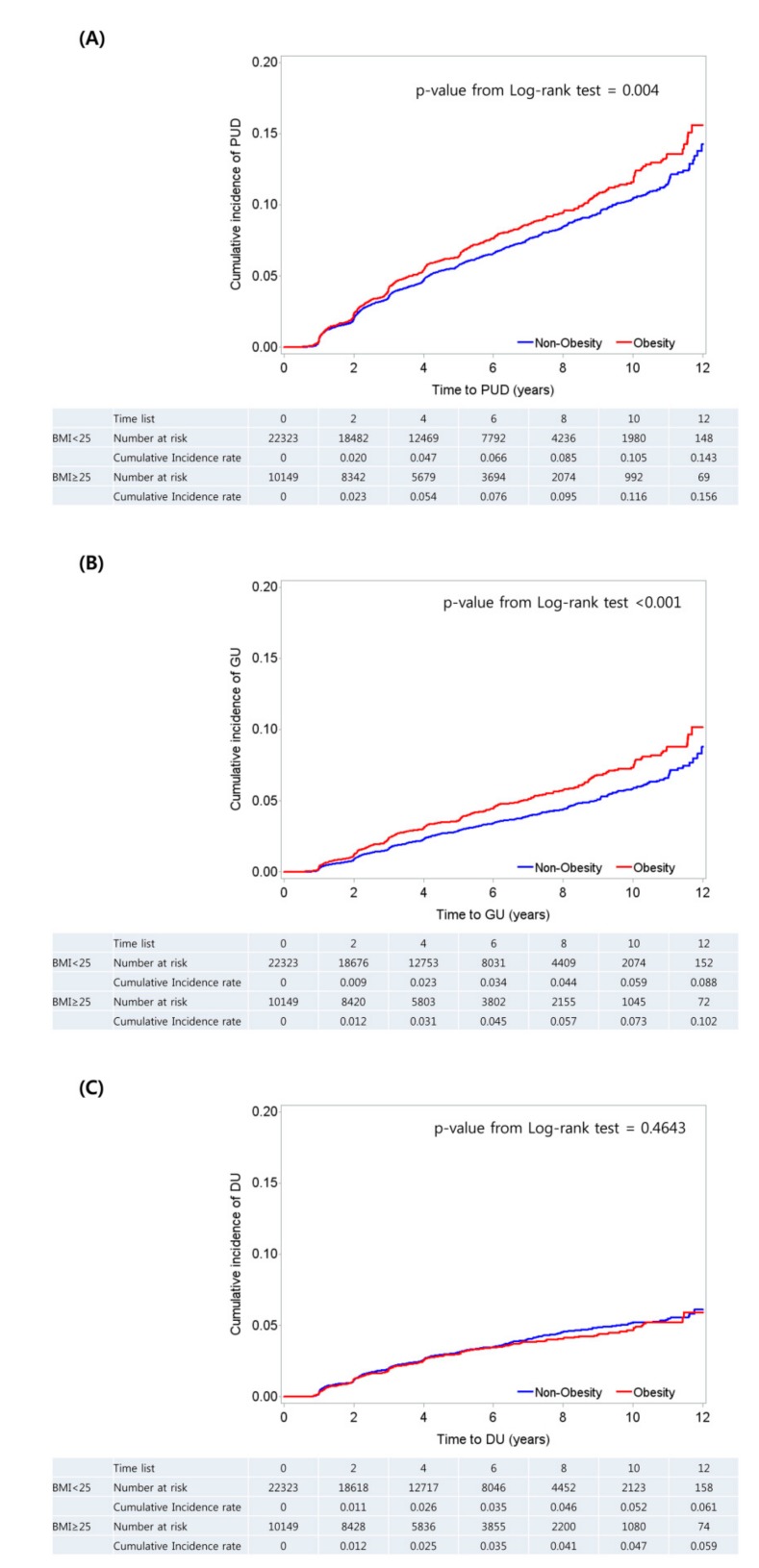
Cumulative incidence of peptic ulcer disease (**A**), gastric ulcer (**B**), and duodenal ulcer (**C**) by obesity status.

Among the 32,472 participants, we identified 1940 cases of PUD (including 1067 cases of GU and 955 cases of DU) by EGD, during 166,256.6 person-years of follow-up. The cumulative incidence rate of PUD and GU were significantly higher in the obese group than the non-obese group (*p* value from Log-rank test = 0.004 and <0.001, respectively). However, the cumulative incidence rate of DU was not significantly different between the two groups (*p* value from Log-rank test = 0.464) (Figure 1).

## 5. Association between Obesity and PUD

Table 2 shows the risk of PUD, GU, and DU. The unadjusted HRs (95% CIs) in the obese group for PUD (1.15 [1.04–1.26]) and GU (1.32 [1.16–1.49]), were significantly higher than in the non-obese group (*p* value = 0.004 and <0.001, respectively). However, the risk of DU in the obese group was not significantly different from the non-obese group (HR 0.95, 95% CI 0.83–1.09, *p* value = 0.469). The HRs (95% CIs) of PUD and GU in the obese group compared to that in the non-obese group in multivariable Model 1 adjusted for age and sex were 0.99 (0.90–1.09), and 1.12 (0.99–1.28), respectively; however, the differences were not significant (*p* value = 0.854 and 0.071, respectively). When obesity was further adjusted for metabolic parameters in Models 2, 3, and 4 (alcohol intake, smoking, physical activity, and blood pressure, use of antihypertensive medication, fasting plasma glucose, use of hypoglycemic medication, triglyceride, HDL-cholesterol, LDL-cholesterol and *Helicobacter pylori* status), PUD and GU consistently showed no association. In contrast, HRs (95% CIs) of DU in the obese group in multivariable Models 1 and 2, 0.83 (0.72–0.96) and 0.81 (0.69–0.97), respectively, were significantly lower compared to that in the non-obese group (*p* value = 0.011 and 0.020, respectively). However, when further adjusted for metabolic parameters in Models 3 and 4, the differences were not statistically significant (*p* value = 0.280 and 0.199, respectively).

## 6. Association between Metabolic Health, Obesity, and PUD

We additionally conducted subgroup analysis considering the metabolic status (Table 3). The unadjusted HRs (95% CI) in the MHO group for PUD and GU of 1.18 (1.03–1.34) and 1.27 (1.07–1.52), were significantly higher compared to those in the MHNO group (*p* value = 0.015 and 0.008, respectively). However, the fully adjusted HRs for metabolic factors were not statistically significant (*p* value = 0.724 and 0.572, respectively); and when adjusted for *Helicobacter pylori*, the HRs were lower (0.83 and 0.89 respectively), with no statistically significant differences (*p* value = 0.136 and 0.447, respectively). The unadjusted HR (95% CI) of MHO for DU was 1.03 (0.86–1.25), but the difference was not significant compared to MHNO (*p* value = 0.732). MHO and DU consistently showed no association when further adjusted for metabolic parameters.

## 7. Discussion

In this study, we found that PUD, particularly GU, had significantly higher cumulative incidence in obese subjects compared to non-obese subjects. There was a significant association between the risk of developing GU and obesity, but when adjusted for possible confounding factors, the association was no more significant. For DU, the cumulative incidence between obese and non-obese groups was not significantly different and there was no significant association. In addition, the risk of developing GU or DU showed no significant associations with MHO.

The association between PUD and obesity was investigated in several studies but the findings remained controversial [4,5,6,7,8,9]. Several studies have reported obesity as an independent risk factor of PUD [4,5,6,7,8]. However, some of these studies had diagnostic limitations, based on self-reported questionnaire for diagnosing PUD [4,6,8]. Thus, less severe cases of PUD could have been excluded, since they have a lesser chance of visits to doctors to undergo EGD or surgery. Of the six studies [4,5,6,7,8,9], only in two studies did participants routinely undergo EGD [7,9]. Furthermore, prior studies were limited by small sample sizes, cross-sectional design, and lack of information on potential confounders [4,5,7,8,9]. Four of the six studies did not adjust for *Helicobacter pylori* status and/or the regular use of either NSAIDs or antiplatelet, which are well-recognized causes of PUD [4,7,8,9]. Interestingly, a recent research using the Korean National Health and Nutrition Examination Survey, showed a contradictory result to this study. BMI was associated with PUD only in men and that high BMI reduced the risk of PUD in men [8]. This study had strength in that it included a large number of subjects (*n* = 23,015) and adjusted nutrients for PUD. However, this was a cross-sectional study, without information about *Helicobacter pylori* or the use of NSAIDs or antiplatelet, and the diagnosis of PUD was based on self-report. These limitations may have led to different conclusions from the same country population. The role of obesity in PUD is not well understood. It is unclear if the reported association is due to elevated BMI in obese subjects and the resultant physiologic stress secondary to nutrient excess or from frequent comorbid illnesses associated with obesity [16]. In contrast, Tsai et al. [9] reported that the increasing trend in BMI effect was not significantly associated with PUD, corresponding to our study results. This study adjusted for age, lifestyle, and sociodemographic factors; and the diagnosis of PUD followed the result of endoscopic inspection in 6318 individuals who visited health screening centers in Taiwan.

In our study, the significant or borderline degree of significant association between obesity and the risk of developing PUD, specifically GU, was found (*p* value <0.001 in the unadjusted model with *p* value of 0.071 for Model 1), but the association disappeared after adjusting for lifestyle factors in Model 2 (*p* value = 0.355). The significant associations between obesity and PUD reported in other studies may have been due to incomplete adjustments for the influence of these lifestyle confounding factors. Moreover, the lack of association between MHO and the risk of GU (*p* value = 0.501 in Model 1) suggests that obesity itself, represented by BMI, had no significant influence on PUD, but the lifestyle factors associated with metabolic syndrome may be more important causative risk factors of PUD [17].

Our study has strength in that the diagnosis of PUD was based on clinical records. Since all subjects underwent EGD, less severe cases without symptoms of PUD were included. In addition to the demographic and lifestyle variables, many metabolic factors, *Helicobacter pylori* status, as well as the regular use of NSAIDs, antiplatelet, or anticoagulants were taken into account. It is well known that obesity accompanies certain medical comorbidities; and much interest has recently focused on obese individuals without metabolic abnormalities, which commonly accompany excess adiposity (a condition known as MHO); however, the prognostic value of MHO is controversial. To the best of our knowledge, no previous research has demonstrated the association between MHO and PUD. In this large cohort study, adjusting for possible influence of confounders, we found that obesity or MHO is not associated with PUD.

There are some limitations to this study. First, the analysis was limited to those who visited the health check-up center, and therefore enrolled subjects were likely to have better economic status than in the general population. Selection bias was possible, although we adjusted for many potential confounders. More severe cases of PUD may have been excluded because usually asymptomatic subjects visit health screening centers. However, subjects enrolled in this study had considerable percentages of those in the active (18.9%) or healing (31.8%) gastric ulcers stages, whereas, 86.0% of those with duodenal ulcer were at the scarring stage. Secondly, *Helicobacter pylori* infection status was not examined for all enrolled subjects.

In conclusion, in this large cohort, the risk of developing PUD was not significantly increased in obese or MHO individuals compared to normal weight individuals. These findings provide compelling comparison evidence with some prior studies. However, previously reported association between obesity and PUD appears to be due to confounding factors including various lifestyle factors or metabolic syndrome. Further prospective investigation is warranted to validate these findings.

## Figures and Tables

**Table 1:** Baseline characteristics of the study subjects according to obesity.

	All	Non-Obese (*n* = 22,323)	Obese (*n* = 10,149)	*p* Value
Age (year)	50.2 ± 8.0	50.0 ± 8.0	50.8 ± 7.9	<0.001
Sex (male, %)	55.7	45.9	77.2	<0.001
Systolic BP (mmHg)	117.1 ± 15.7	114.8 ± 15.4	122.1 ± 15.0	<0.001
Diastolic BP (mmHg)	73.2 ± 11.2	71.5 ± 11.0	76.9 ± 10.6	<0.001
Waist circumference (cm)	82.8 ± 9.1	78.8 ± 7.2	91.4 ± 6.7	<0.001
Current smoker (%)	20.8	17.2	28.8	<0.001
Heavy drinker (%)	26.0	21.3	36.4	<0.001
Regular exercise (%)	45.4	45.1	46.1	<0.001
Hypertension (%)	17.9	13.0	28.6	<0.001
Diabetes (%)	6.7	5.5	9.2	<0.001
Total cholesterol	196.5 ± 33.9	195.1 ± 33.4	199.7 ± 35.0	<0.001
LDL-cholesterol	125.2 ± 30.3	122.9 ± 30.0	130.2 ± 30.5	<0.001
HDL-cholesterol	56.8 ± 15.0	59.8 ± 15.3	50.3 ± 12.1	<0.001
Triglycerides	122.6 ± 78.4	108.4 ± 66.0	154.0 ± 93.0	<0.001
Fasting blood glucose	94.7 ± 17.6	92.5 ± 15.9	99.4 ± 20.1	<0.001
Insulin	7.7 ± 4.4	6.8 ± 3.6	9.8 ± 5.1	<0.001
HOMA-IR	1.7 ± 1.1	1.4 ± 0.8	2.2 ± 1.4	<0.001
hsCRP	0.1 ± 0.3	0.1 ± 0.3	0.2 ± 0.4	<0.001
PUD	6.0	6.0	6.6	0.001
GU	3.3	3.0	4.0	<0.001
DU	3.0	3.0	2.9	0.596
*Helicobacter pylori status (%, in 1671 subjects)*	41.9	42.9	40.1	0.281

BP, blood pressure; LDL, low-density lipoprotein; HDL, high-density lipoprotein; hsCRP, high-sensitivity C-reactive protein, HOMA-IR, homeostatic model assessment-insulin resistance; PUD, peptic ulcer disease; GU, gastric ulcer; DU, duodenal ulcer.

**Table 2:** Association between obesity and the risk of peptic ulcer disease.

	Cases/*n* (%)	Model 1		Model 2		Model 3		Model 4	
		HR (95% CI)	*p* Value	HR (95% CI)	*p* Value	HR (95% CI)	*p* Value	HR (95% CI)	*p* Value
PUD			0.853		0.511		0.922		0.610
Non-Obese	1270 (5.7)	1.00		1.00		1.00		1.00	
Obese	670 (6.6)	0.99 (0.90–1.09)		0.96 (0.86–1.08)		1.01 (0.86–1.19)		0.96 (0.80–1.14)	
GU			0.071		0.355		0.299		0.789
Non-Obese	664 (3.0)	1.00		1.00		1.00		1.00	
Obese	403 (4.0)	1.12 (0.99–1.28)		1.08 (0.92–1.25)		1.12 (0.91–1.38)		1.03 (0.83–1.28)	
DU			0.011		0.020		0.280		0.199
Non-Obese	664 (3.0)	1.00		1.00		1.00		1.00	
Obese	291 (2.9)	0.83 (0.72–0.96)		0.83 (0.69–1.00)		0.87 (0.68–1.12)		0.83 (0.63–1.10)	

Model 1: adjusted for age, sex; Model 2: adjusted for variables in model 1, plus drinking, smoking, and physical activity); Model 3: adjusted for variables in model 2, plus blood pressure, use of antihypertensive medications, fasting blood glucose, use of hypoglycemic medications, triglyceride, HDL-cholesterol, and LDL-cholesterol; Model 4: adjusted for variables in model 3, plus *Helicobacter pylori* status. PUD, peptic ulcer disease; GU, gastric ulcer; DU, duodenal ulcer; HR, hazard ratio; CI, confidence interval.

**Table 3:** Association between metabolic health, obesity, and peptic ulcer disease.

	Cases/*n* (%)	Model 1		Model 2		Model 3		Model 4	
		HR (95% CI)	*p* Value	HR (95% CI)	*p* Value	HR (95% CI)	*p* Value	HR (95% CI)	*p* Value
PUD									
metabolically healthy, non-obese	922 (5.4)	1.00		1.00		1.00		1.00	
metabolically unhealthy, non-obese	348 (6.5)	1.02 (0.90–1.16)	0.777	0.98 (0.84–1.14)	0.752	1.02 (0.80–1.28)	0.895	0.90 (0.69–1.18)	0.443
metabolically healthy, obese	302 (6.7)	1.00 (0.88–1.14)	0.983	0.95 (0.81–1.11)	0.519	1.04 (0.83–1.30)	0.764	0.83 (0.65–1.06)	0.26
metabolically unhealthy, obese	368 (6.6)	0.93 (0.99–0.88)	0.93	0.96 (0.82–1.12)	0.594	0.99 (0.77–1.28)	0.949	1.00 (0.76–1.32)	0.99
GU									
metabolically healthy, non-obese	470 (2.8)	1.00		1.00		1.00		1.00	
metabolically unhealthy, non-obese	194 (3.6)	1.05 (0.89–1.25)	0.566	1.03 (0.84–1.25)	0.803	1.06 (0.78–1.44)	0.715	0.86 (0.62–1.20)	0.365
metabolically healthy, obese	168 (3.7)	1.06 (0.89–1.27)	0.501	1.01 (0.81–1.25)	0.95	1.09 (0.81–1.47)	0.523	0.89 (0.65–1.21)	0.447
metabolically unhealthy, obese	235 (4.2)	1.21 (1.03–1.42)	0.023	1.15 (0.95–1.40)	0.165	1.20 (0.87–1.66)	0.264	1.04 (0.74–1.46)	0.825
DU									
metabolically healthy, non-obese	495 (2.9)	1.00		1.00		1.00		1.00	
metabolically unhealthy, non-obese	169 (3.1)	1.07 (0.90–1.28)	0.816	0.91 (0.73–1.13)	0.384	0.91 (0.64–1.29)	0.583	0.80 (0.53–1.22)	0.295
metabolically healthy, obese	142 (3.1)	1.03 (0.86–1.25)	0.249	0.85 (0.67–1.07)	0.164	0.95 (0.69–1.32)	0.771	0.83 (0.56–1.21)	0.324
metabolically unhealthy, obese	149 (2.7)	0.91 (0.76–1.09)	0.007	0.74 (0.59–0.94)	0.012	0.73 (0.50–1.07)	0.109	0.69 (0.45–1.08)	0.102

Model 1: adjusted for age, sex; Model 2: adjusted for variables in model 1, plus drinking, smoking, and physical activity; Model 3: adjusted for variables in model 2, plus blood pressure, use of antihypertensive medications, fasting blood glucose, use of hypoglycemic medications, triglyceride, HDL-cholesterol, LDL-cholesterol; Model 4: adjusted for variables in model 3, plus *Helicobacter pylori* status. PUD, peptic ulcer disease; GU, gastric ulcer; DU, duodenal ulcer; HR, hazard ratio; CI, confidence interval.

## References

[1] Malfertheiner P., Chan F.K., McColl K.E. (2009). Peptic ulcer disease. Lancet.

[2] Yuan Y., Padol I.T., Hunt R.H. (2006). Peptic ulcer disease today. Nat. Clin. Pract. Gastroenterol. Hepatol..

[3] Quan C., Talley N.J. (2002). Management of peptic ulcer disease not related to helicobacter pylori or nsaids. Am. J. Gastroenterol..

[4] Garrow D., Delegge M.H. (2010). Risk factors for gastrointestinal ulcer disease in the us population. Dig. Dis. Sci..

[5] Aro P., Storskrubb T., Ronkainen J., Bolling-Sternevald E., Engstrand L., Vieth M., Stolte M., Talley N.J., Agreus L. (2006). Peptic ulcer disease in a general adult population: The kalixanda study: A random population-based study. Am. J. Epidemiol..

[6] Boylan M.R., Khalili H., Huang E.S., Chan A.T. (2014). Measures of adiposity are associated with increased risk of peptic ulcer. Clin. Gastroenterol. Hepatol..

[7] Wang F.W., Tu M.S., Mar G.Y., Chuang H.Y., Yu H.C., Cheng L.C., Hsu P.I. (2011). Prevalence and risk factors of asymptomatic peptic ulcer disease in Taiwan. World J. Gastroenterol..

[8] Lee B.J., Kim J., Kim K.H. (2018). Association of gastric and duodenal ulcers with anthropometry and nutrients: Korean national health and nutrition examination survey (knhanes ii–iv) 2001–2009. PLoS ONE.

[9] Tsai W.L., Yang C.Y., Lin S.F., Fang F.M. (2004). Impact of obesity on medical problems and quality of life in Taiwan. Am. J. Epidemiol..

[10] Sims E.A. (2001). Are there persons who are obese, but metabolically healthy?. Metab. Clin. Exp..

[11] Munoz-Garach A., Cornejo-Pareja I., Tinahones F.J. (2016). Does metabolically healthy obesity exist?. Nutrients.

[12] (2004). Appropriate body-mass index for asian populations and its implications for policy and intervention strategies. Lancet.

[13] Wildman R.P., Muntner P., Reynolds K., McGinn A.P., Rajpathak S., Wylie-Rosett J., Sowers M.R. (2008). The obese without cardiometabolic risk factor clustering and the normal weight with cardiometabolic risk factor clustering: Prevalence and correlates of 2 phenotypes among the us population (nhanes 1999–2004). Arch. Int. Med..

[14] Hsu P.I., Lai K.H., Tseng H.H., Lo G.H., Lo C.C., Lin C.K., Cheng J.S., Chan H.H., Ku M.K., Peng N.J. (2001). Eradication of helicobacter pylori prevents ulcer development in patients with ulcer-like functional dyspepsia. Aliment. Pharmacol. Ther..

[15] Matthews D.R., Hosker J.P., Rudenski A.S., Naylor B.A., Treacher D.F., Turner R.C. (1985). Homeostasis model assessment: Insulin resistance and beta-cell function from fasting plasma glucose and insulin concentrations in man. Diabetologia.

[16] Wisse B.E., Kim F., Schwartz M.W. (2007). Physiology. An integrative view of obesity. Science.

[17] Watanabe S., Hojo M., Nagahara A. (2007). Metabolic syndrome and gastrointestinal diseases. J. Gastroenterol..

